# Growth hormone-releasing hormone receptor (GHRH-R) and its signaling

**DOI:** 10.1007/s11154-025-09952-x

**Published:** 2025-02-12

**Authors:** Gabor Halmos, Zsuzsanna Szabo, Nikoletta Dobos, Eva Juhasz, Andrew V. Schally

**Affiliations:** 1 Department of Biopharmacy, Faculty of Pharmacy, University of Debrecen, Rex u. 10, Debrecen, 4032 Hungary; 2 Veterans Affairs Medical Center, Endocrine, Polypeptide and Cancer Institute, Miami, FL 33125 USA; 3 Department of Pediatrics, Faculty of Medicine, University of Debrecen, Debrecen, 4032 Hungary; 4 Department of Pathology, Department of Medicine, Divisions of Hematology-Oncology and Endocrinology, Miller School of Medicine, University of Miami, Miami, FL 33101 USA; 5 Sylvester Comprehensive Cancer Center, University of Miami, Miami, FL 33136 USA

**Keywords:** Growth hormone-releasing hormone (GHRH), GHRH receptor (GHRH-R), Splice variants (SVs), Signalization

## Abstract

The hypothalamic polypeptide growth hormone-releasing hormone (GHRH) stimulates the secretion of growth hormone (GH) from the pituitary through binding and activation of the pituitary type of GHRH receptor (GHRH-R), which belongs to the family of G protein-coupled receptors with seven potential membrane-spanning domains. Various splice variants of GHRH-R (SV) in human neoplasms and other extrapituitary tissues were demonstrated and their cDNA was sequenced. Among the SVs, splice variant 1 (SV1) possesses the greatest similarity to the full-length GHRH-R and remains functional by eliciting cAMP signaling and mitogenic activity upon stimulation by GHRH. In this review, we briefly discuss the activation, regulation, molecular mechanisms and signaling pathways of GHRH-Rs and their SVs in various tissues and also summarize the expression, biological activities and potential function of GHRH, its analogs and their receptors. A large body of work have extensively studied and evaluated potential clinical applications of agonists and antagonists of GHRH in diverse fields, including oncology, endocrinology, obesity, diabetes, other metabolic dysfunctions, cardiology, immune functions, mood disorders, Alzheimer’s and lung disease, ophthalmology, inflammation, wound healing and other applications. These results strongly support the potential therapeutic use of GHRH analogs in human medicine in the near future.

## General overview of growth hormone-releasing hormone (GHRH) and its receptors (GHRH-R)

### The structure of G-protein–coupled receptors and GHRHs

G-protein-coupled receptors (GPCRs) consists of more than 800 receptors composing the largest family of proteins in humans. GPCRs are membrane proteins constituting of seven transmembrane helices (TM1–TM7) and three extracellular (ECL1–ECL3) and three intracellular (ICL1–ICL3) loops. The GPCR superfamily includes several subfamilies; Class A rhodopsin-like, Class B secretin-like, Class C metabotropic glutamate/pheromone, Class F frizzled (FZD), Taste receptors (TAS1R, TAS2R), Vomeronasal receptors (VN1R, VN2R) and 7TM orphan receptors. Class B GPCRs play a key role in hormonal homeostasis and acts as valuable drug targets for endocrine and neurological disorders. Growth hormone-releasing hormone receptor (GHRH-R) belongs to class B GPCRs, and physiologically mainly expressed on the growth-stimulating somatotropic cells in the pituitary gland [1–4]. The human pituitary type GHRH-R (pGHRH-R) gene is localized at human chromosome 7p 14e15 and the protein consists of 423 amino acids. Characterization of the genomic sequence revealed that the human GHRH-R gene consists of 13 exons and spans 15 kilobases [5–9].

### The presence of GHRH-R in human tissues

GHRH-R is primarily expressed in the anterior pituitary gland, however, it varies with different developmental stages and the number of GHRH-R decreases during aging [3, 10–13]. GHRH-R is also present in several other human tissues, such as myocardium, lymphocytes, testes, ovaries, skin, placenta, kidney and pancreas and is involved in different biological, physiological and pathological processes [3, 10, 12, 14]. Furthermore, the presence of GHRH-Rs has been shown in primary human tumor cells, representing a target for diagnostic or therapeutic intervention using GHRH analogs [3, 12, 13].

The effects of GHRH and its analogs are mediated by pGHRH-R [2, 3, 9, 10, 15, 16]. Activation of GHRH-R upon the binding of GHRH, a 44-amino acid peptide released by the hypothalamus, results in the secretion and production of GH through cyclic adenosine monophosphate (cAMP)-dependent pathways [3, 14, 16, 17]. The 44-amino acid forms of GHRH were first isolated and identified from human pancreatic tumors and only subsequently purified from hypothalamic tissue [18–20]. The full biological activity is contained in the N-terminal 29 amino acid sequence [GHRH(1–29)NH2] [3, 10, 12, 15, 21].

(Fig. 1.)

**Fig. 1:**
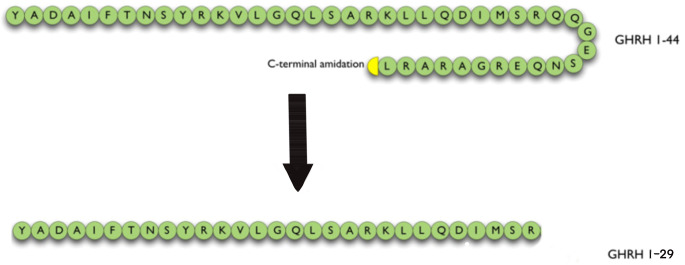
Amino acid sequences of human GHRH (GHRH 1–44). The full biological activity is retained in the N-terminal 29 amino acid sequence [GHRH(1–29)NH_2_]

### Splicing and splice variants of GHRH-R

GPCRs comprise the major group of cellular receptors and the largest protein superfamily in the human genome. Alternative splicing, e.g. exon skipping, splice site selection, and intron retention, resulting in deletion, exchange, and insertion of receptor sequences is often observed among GPCRs [1, 7, 9, 16]. Also, splice variants of many GPCRs, including GHRH-Rs, have been shown in different cancer types, however, their biological significance is poorly understood. Alternative splice variants of GHRH-Rs have been found under hypoxic microenvironment in solid tumors, suggesting a specific role in cellular adaptation and malignant processes [1, 9].

Rekasi et al. in 2000 detected two major splice variants (SVs) of the GHRH-R in human extrapituitary cells as well as in human cancer cell lines [2, 22, 23]. Furthermore, four truncated GHRH-R SVs have been observed in nonmalignant human tissues, numerous different types of human cancer specimens and various human cancer cell lines [22–24]. Thus, pGHRH-R and its SVs are considered as potential targets for antitumor therapy due to the antiproliferative effect of GHRH antagonists [3, 11, 15, 24–27].

## Overview of GHRH and GHRH-R signaling

### Expression and signaling of GHRH-R and SVs in different tissues

Healthy human tissues as well as human tumors express pGHRH-Rs and its splice variants both at mRNA and protein level. The expression of pGHRH-R mRNA and its protein products was demonstrated in normal human pituitary, kidney, lung, prostate, and liver [12, 24].

These results strengthens and magnifies the hypothesis that GHRH and its receptors have a fundamental role in the pathophysiology of several human cancers. Using real-time PCR, Western blotting, and radioligand-binding assays, the mRNA and protein expression of GHRH-R, SV1 and immunoreactive GHRH have been observed in different human cancer cell lines and in human lung cancers, lymphomas, pancreatic cancers, glioblastomas, small-cell lung carcinomas, and in human nonmalignant prostate, liver, lung, kidney, and pituitary [12, 24, 28–31]. Moreover, non-Hodgkin’s lymphomas, DBTRG-05 glioblastoma, healthy specimens of human kidney, liver, lung, prostate, and pituitary tissues expressed all SVs at mRNA level [10, 12, 24, 32]. Additionally, SV1 expression has also been found in the cytoplasm in primary endometrial carcinoma [10]. These specimens also showed GHRH expression, suggesting an autocrine stimulatory loop between GHRH and the SV1 of the GHRH-R. Comparable interaction of SV1 and GHRH was also described in prostate carcinoma [3, 10, 11].

Cytoplasmic supranuclear SV1 expression has been observed both in healthy colon mucosa and human colorectal cancer [31]. Since SV1 is a membrane receptor, it can also be activated by other peptides having similar structure to GHRH, e.g. VIP or PACAP. Neoplasms also showed cytoplasmic expression of SV1. Well-differentiated colorectal carcinomas and those confined to the mucosa and submucosa showed strong expression of SV1, while low-differentiated tumors and those with pericolic fat invasion express it in a much lower amount. Nonmetastatic tumors show elevated SV1 expression compared to colorectal cancers with liver metastasis. Also, increased SV1 expression was related to favorable prognosis and better survival of patients with colorectal cancer [29, 31].

Mezey et al. in 2014 reported the presence of SV1 in human glioblastoma. Interestingly, they found a negative correlation between the expression of GHRH and SV1 genes and the prognosis of glioblastoma; the higher expression correlates with poorer prognosis [30]. Also, GHRH positive, but SV1 negative cases showed better overall survival. These results have partially been confirmed by Farkas et al. (2012) who showed GHRH-R expression and poor response of rectal cancers to chemotherapy [29]. These findings support the hypothesis that tumor progression can be affected by paracrine and autocrine GHRH release and tumoral GHRH expression can be decreased by autoregulating factors of the tumor. The down-regulation of GHRH expression through SV1 or other receptors mediated by systemic GHRH or other ligands can result in negative feedback mechanisms leading to decreased autocrine GHRH release. GHRH can also act on other receptors leading to protective effects [30].

In conclusion, both pGHRH-R and SV1 can be found in healthy and neoplastic human tissues, mediating GHRH signaling. The expression of GHRH receptors has been found in primary human prostatic, breast, endometrial, lung, adrenal carcinomas and uveal melanomas, as well as in experimental human cell lines of virtually all major types of malignancies, including prostatic, ovarian, breast, endometrial, lung (SCLC and non-SCLC), colorectal, gastric, pancreatic, renal, glioblastomas, osteogenetic and Ewing sarcomas, lymphomas, and uveal melanomas [3, 10, 11, 14, 15, 23, 26, 33–37]. Collectively, these data suggest that in different human tumors, GHRH and its tumoral receptors might form an autocrine/paracrine mitogenic loop involved in tumor development and progression.

### Regulation, signaling mechanism and pathways of GHRH-R

GHRH peptide, secreted by the hypothalamus, stimulates the synthesis and release of GH from the pituitary gland. GHRH is initially synthesized as a preprohormone containing 108 amino acids. Mature GHRH carries 44 amino acids, this molecule is formed from the precursor molecule when the N-terminal end is enzymatically cleaved to form a C-terminal GHRH-bound peptide molecule (GHRH-RP) [16, 38–40].

GHRH acts as an autocrine/paracrine growth factor in many tumors [38, 39]. GHRH is expressed in the limbic system, cerebral cortex, posterior part of the brain, peripheral nervous system, gastrointestinal tract, pituitary, gonads, adrenal glands, thyroid, lung and kidney [6, 38, 39].

GH produced by pituitary cells also stimulates the production of insulin-like growth factor I (IGF-I) in the liver [38, 39]. The activation of GHRH-R results in the secretion and production of GH via cyclic adenosine monophosphate (cAMP)-dependent pathways [16, 41]. Uninterrupted or recurring stimulation of GHRH-R in the pituitary leads to attenuation of GH release [42].

**Fig. 2:**
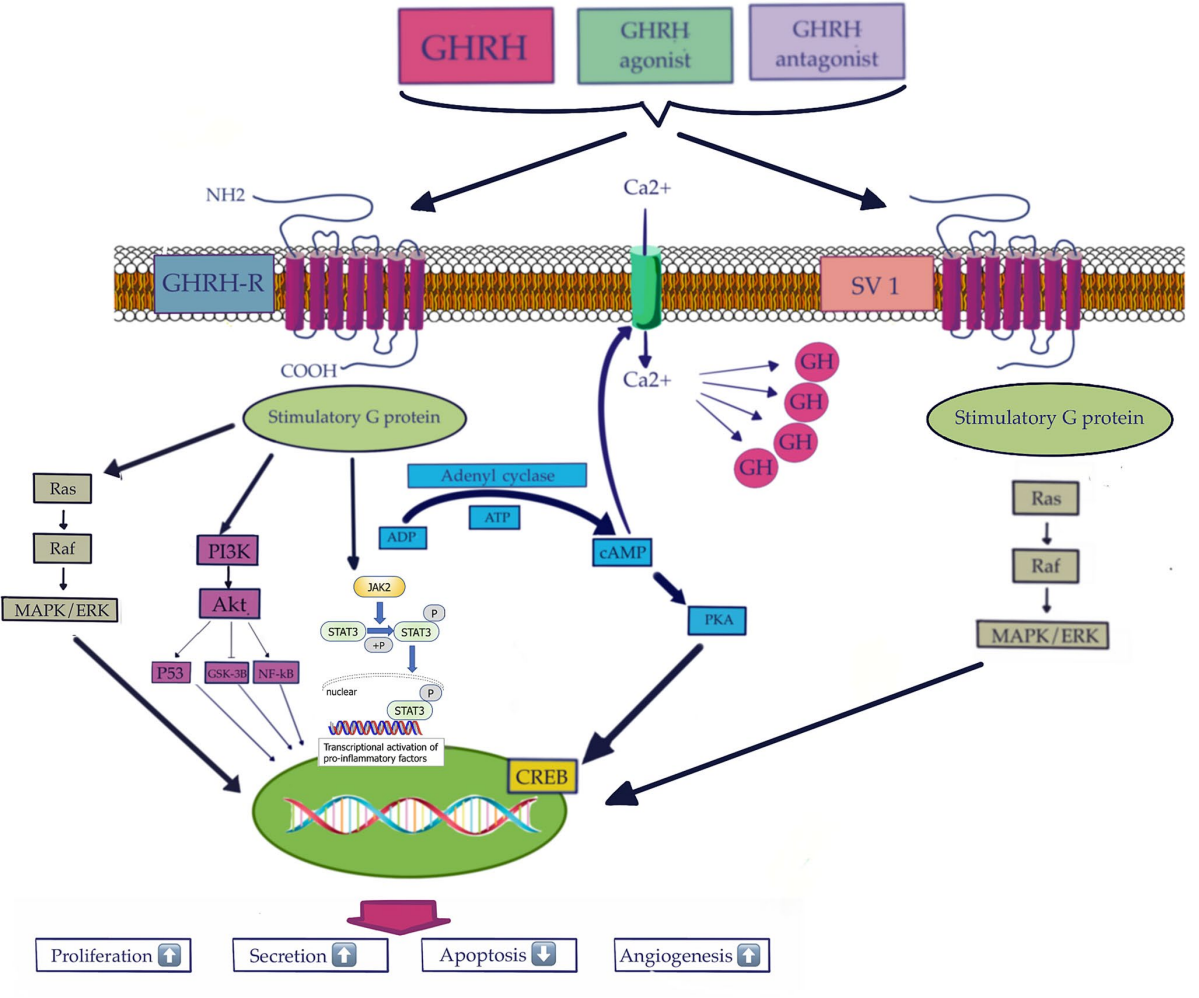
Schematic drawing and summary diagram of the potential role and signaling mechanisms, cascades and cellular effects of GHRH, GHRH agonists and GHRH antagonists mediated by GHRH-R and SV1

After GHRH binds to GHRH-R, GHRH-R activates a G protein by catalyzing the binding of guanosine-5-triphosphate (GTP) to the α-subunit on the intracellular side [38]. Receptor desensitization is the main mechanism responsible for GH response attenuation and it involves the uncoupling of the G protein that activates the adenylate cyclase [42]. The activation of adenylate cyclase increases cAMP concentration, resulting in an increase in Ca^2+^ ions, and consequent GH release into the systemic circulation. The increase in cAMP results in activation of the protein kinase A pathway, determining cell proliferation, and GH and GHRH-R synthesis [38, 42].The subsequent process results in the opening of a sodium channel, which leads to its depolarization. As a result a voltage-dependent Ca^2+^ channel will open, allowing calcium influx, which directly causes the release of GH stored in secretory granules [6, 9, 43]. (Fig. 2.)

cAMP also binds and activates the regulatory subunits of protein kinase A (PKA), which phosphorylate and activate the transcription factor CREB protein, the binding protein of the cAMP response element [6, 9, 38, 43].

CREB may induce the synthesis of pituitary-specific transcription factor Pit-1, and an increase in Pit-1 may lead to a subsequent increase in GH gene expression, ultimately replenishing the cellular stores of GH so that the pituitary somatotroph can respond appropriately to the next pulse of GHRH [38, 44]. Phosphorylated CREB, together with its coactivators p300 and CREB-binding protein, enhances GH transcription by binding to CRE cAMP response elements in the promoter region of the GH gene. CREB also stimulates GHRH-R gene transcription. In the phospholipase C pathway. Activation of phospholipase C (PLC) produces diacylgycerol (DAG) and inositol triphosphate (IP3), IP3 induces the release of intracellular Ca^2+^ from the endoplasmic reticulum, increasing the cytosolic Ca^2+^ concentration, resulting in vesicle fusion and release of secretory vesicles containing preformed GH [38, 44]. (Fig. 2.).

MAP kinase activation and ERK phosphorylation have also been reported in the pituitary in a cAMP/PKA/PKC-dependent manner. GHRH can stimulate Ras/MAPAK through big subunits to promote cell growth [6, 9, 38].

Some studies have also shown that the inhibition of apoptosis in the myocardium, which is mediated by the GHRH-R involves the modulation of ERK1 and ERK2 and PI3K_Akt signaling. This became evident because ERK1/2- and PI3K/Akt-specific inhibitors abolish these effects [6, 9, 38].

Although the adenylate cyclase–protein kinase A pathway is the principal transduction mechanism that mediates GHRH actions on somatotropic cells, other mechanisms, such as the inositol phosphate–diacylglycerol–protein kinase C system, the Ca^2+^- calmodulin system, and the arachidonic acid–eicosanoid system are also believed to play a minor role. Uninterrupted or recurring stimulation of GHRH-R in the pituitary leads to attenuation of GH release [38, 42].

GHRH-R also causes the activation of the phospholipase C pathway (IP3/DAG pathway) and some other smaller pathways, through which it also induces the production of GH [38].

GHRH-R with different C-terminals binds and activates different types of G proteins, thereby activating different intra-cellular signaling pathways. For example, GHRH-R can mediate the cAMP/PKA/CREB signaling pathway when it couples to Gs-type G protein, whereas it can activate the NOS/NO/cGMP signaling pathway when it couples to Go-type G protein [45–47].

### Conformational changes during the activation of GHRH-R

During the activation of the GHRH-R, conformational changes also occur in the structure of the receptor, which help the binding of the receptor to the connection of an intracellular G-head and thereby activate pathways linked to G-proteins via cAMP. This structural change means that the C-terminal α-helix of the GHRH-R recognizes and binds to the extracellular domain, thereby allowing its N-terminus to interact with the extracellular TM core. This is followed by a major conformational change involving a large kink in TM6 to open the intracellular face for G-protein coupling [16, 42].

### Factors mediating GHRH-R signaling

GH is one of the major pituitary hormones and critical regulator of organism growth and metabolism, and it is also needed for optimal immune cell function. Lipopolysaccharide (LPS) and LPS-induced cytokines can directly stimulate GH secretion from somatotroph cells [48–50]. GHRH-R, as the most important GPCR in regulating GH release, is reportedly stimulated by LPS. In addition, the SVs of GHRH-R are also regulated by LPS [38, 51]. In several preliminary studies, the response of GHRH-R SVs and GH to LPS was examined. LPS induced GH and GHRH-R expression but reduced GHRH-R SV1 and SV2 expressions. The findings indicated that the different GHRH-R SVs would display distinct expression levels under the stimulation of certain factors [51].

### Signaling mechanisms of GHRH-R in LPS-induced acute ocular inflammation

As mentioned before, emerging evidence indicates that GHRH-R is involved in a wide spectrum of extra-pituitary activities, including tumor growth and inflammation. The molecular mechanism of inflammatory processes in acute ocular inflammation is also most likely mediated by GHRH-R. In human ciliary epithelial cells LPS elevates the expression of the GHRH-R gene through the phosphorylation of NF-κB that will lead to the activation of the JAK2/STAT3 pathway, leading to cytokine and chemokine production [38, 52–54].

### Pituitary miRNAs target GHRH-R SVs to regulate GH synthesis by mediating different intracellular signaling pathways

It has been reported that miRNA also can mediate GH synthesis by regulating GHRH-R SVs. Pituitary miRNAs (i.e., let-7e and miR-328-5p), control GH synthesis by targeting different SVs of GHRH-R. The response of let-7e and miR-328-5p to GHRH was also shown, which proved that let-7e and miR-328-5p are involved in GH synthesis mediated by GHRH-R [51]. Accordingly, GHRH promotes the expression of GH and GHRH-R SVs. Both let-7e and miR-328-5p inhibited GH expression and regulated GHRH-R by targeting different GHRH-R SVs. Interestingly, let-7e significantly increased under the action of GHRH, whereas miR-328-5p significantly decreased at high GH expression [51]. Based on the literature it is suggested that the response of miRNAs to the regulators may depend on acting time and dose. It is also assumed that let-7e plays a major inhibitory role in regulating GH synthesis, whereas miR-328-5p maintains the dynamic balance. GHRH-R with different C-terminals binds and activates different types of G proteins, thereby activating different intracellular signaling pathways. As mentioned earlier, GHRH-R can mediate the cAMP/PKA/CREB signaling pathway when it couples to Gs-type G protein, whereas it can activate the NOS/NO/cGMP signaling pathway when it couples to Go-type G protein. According to the literature, let-7e was involved in the NOS/NO signaling pathway and miR-328-5p contributed more to the PKA/CREB signaling pathway. Through vector based transfection it was proved that the protein coded by the GHRH-R transcript regulates GH through the NOS/NO signaling pathway, whereas the GHRH-R coded by GHRH-R SV1and SV2 regulates GH through the PKA/CREB signaling pathway [38, 42, 45, 46, 51].

### The role of GHRH agonists and antagonists in health, diseases and therapy via expression, regulation and signaling of GHRH-R and its SVs

GHRH regulates the secretion of GH, which virtually controls metabolism and growth of every tissue through its binding to the GHRH-R. Dysfunction in GHRH-R signaling is associated with abnormal growth, making GHRH-R an attractive therapeutic target against dwarfism (e.g., isolated GH deficiency, IGHD), gigantism, lipodystrophy and certain cancers. GHRH forms an extensive and continuous network of interactions involving all the extracellular loops (ECLs), all the transmembrane (TM) helices except TM4, and the extracellular domain (ECD) of GHRHR, especially the N-terminus of GHRH that engages a broad set of specific interactions with the receptor.

Over the past two decades several highly potent GHRH agonists of JI and MR class were synthesized and evaluated biologically [3, 14, 21, 26, 43, 55–59].

A large body of work shows that GHRH agonists, such as MR-409, improve pancreatic β-cell proliferation and metabolic functions and facilitate engraftment of islets after transplantation in rodents. Accordingly, GHRH agonists offer a new therapeutic approach to treating diabetes. Various studies demonstrate that GHRH agonists promote repair of cardiac tissue, producing improvement of ejection fraction and reduction of infarct size in rats, reduction of infarct scar in swine, and attenuation of cardiac hypertrophy in mice, suggesting clinical applications [56]. The presence of GHRH-Rs in ocular tissues and neuroprotective effects of GHRH analogs in experimental diabetic retinopathy indicates their possible therapeutic applications for eye diseases [3, 38, 60].

Other effects of GHRH agonists, include acceleration of wound healing, activation of immune cells, anti-inflammatory properties and action on the central nervous system and in neurodegenerative conditions [3, 14, 38, 61–64].

In vitro, GHRH agonists can stimulate growth of human cancer cells and upregulate GHRH-Rs. However, in vivo, GHRH agonists inhibit growth of human cancers xenografted into nude mice and downregulate pituitary and tumoral GHRH-Rs [21]. These various potential beneficial effects of GHRH analogs are possible because of the presence of GHRH-Rs in many cells and tissues. We can no longer ignore the many possible clinical applications of GHRH agonistic analogs, the list of which is continuously extending [21]. Therefore, GHRH and its agonistic analogs, including tesamorelin, MR-409 and JI-38 have been developed as potential therapeutic agents to treat diabetes, cancers, cardiovascular and other diseases [16].

In the endeavor to explore novel methods for treatment of cancer and other malignancies, a large number of potent analogs of GHRH have been designed, synthetized, and developed. Over the past 2 decades, many advanced and powerful agonists and antagonists of GHRH with high receptor affinity have been developed [3, 11, 21, 33, 38, 65]. The development of GHRH antagonists was started after it was established that somatostatin analogs do not adequately suppress GH and IGF-I levels in patients with tumors dependent on IGF-I [2, 15]. It was found that replacement of Ala2 by D-Arg2 in GHRH(1–29)NH2 resulting in [Ac-Tyr1,D-Arg2]hGHRH(1–29)NH2 generates GHRH antagonism [41]. Systematic efforts to develop better GHRH antagonists led to antagonists MZ-4-71 and MZ-5-156 [26, 38, 65]. Other substitutions were then incorporated into GHRH analogs yielding antagonists such as JV-1-36, JV-1-38, and JV-1-65 which all manifested increased inhibitory activity [2, 26]. During the past two decades we have synthesized several hundred new antagonists of GHRH, incorporating various substitutions with less common non-coded amino acids and other changes. This work generated new highly potent GHRH antagonists such the MZ-J, JMR, MIA and AVR series of improved GHRH antagonists [26]. We are now engaged in an active selection of the antagonist candidate best suited for clinical development.

G protein-coupled peptide hormone receptors represent an important family of molecular targets in cancer. Many of them show significant overexpression in tumors. Radiolabeled receptor ligands can be applied to targeted imaging and radiotherapy of receptor-expressing tumors. Moreover, peptide hormone analogs that interfere with receptor-regulated tumor cell functions, such as hormone secretion and proliferation, are used for therapy. In the last few years, it has increasingly been recognized that tumoral peptide receptors correspond not only to the wild-type forms, but also to splice variants [38]. The functional characteristics of these splice variants often differ substantially from those of their wild-type counterparts. While some splice variants inhibit expression or ligand binding and signaling of the corresponding wild-type forms, others amplify wild-type effects. Peptide receptor splice forms may thus influence clinical applications based on wild-type receptor expression and functions. Furthermore, they also represent potential clinical targets on their own [38, 66]. Kiaris et al. reported that the expression of SV1 augments the stimulatory responses to GHRH(1–29)NH2 or GHRH agonist JI-38 and inhibitory responses to GHRH antagonist JV-1-38 as compared with pcDNA3 controls [34]. The stimulation of SV1-expressing cells by GHRH or JI-38 is followed by an increase in cAMP production, but no GH release occurs. VIP had no effect, and its antagonist JV-1-53 did not inhibit the proliferation of SV1-expressing cells stimulated by GHRH. This result suggest that SV1 could mediate responses of nonpituitary cells and various tumors to GHRH and GHRH antagonists. The presence of SV1 in several human cancer models provides a rationale for antitumor therapy based on the blockade of this receptor by specific GHRH antagonists [34].

Although considerable structural homology exists between GHRH and VIP peptides as well as between their receptors, Halmos et al., 2000 showed that the antiproliferative effect of GHRH antagonists such as JV-1-36, JV-1-38, and JV-1-42 is exerted through a mechanism independent of VIP receptors [22]. Though high-affinity receptors for VIP are also present on CAKI-1 renal cell carcinoma (RCC), it was clearly demonstrated that these receptors do not recognize hGHRH and its analogs and display markedly lower or almost negligible binding to GHRH antagonists such as JV-1-42. This finding suggest that endocrine action of GHRH antagonists based on inhibition of GH/IGF-I axis, antitumor effects of these analogs can be exerted directly through GHRH-Rs expressed by CAKI-1 RCC [11, 22, 55, 65]. GHRHR antagonists strongly augment apoptosis and decrease proliferation of multiple types of cancer cells in vitro and in vivo.

Consequently, the tumor inhibitory effects of GHRH antagonists in 16 types of human cancers, represented by nearly 50 human cancer lines including prostatic, breast, ovarian, renal, gastric, pancreatic, lung, and recently, acute myeloid leukemia appear to be based in part on the interference with the local stimulatory GHRH system. Thus, GHRH antagonists can directly block the tumoral receptors for GHRH and prevent the activation of the autocrine/paracrine GHRH in cancers. In addition, the production of tumoral IGF-I and IGF-II also appears to be inhibited directly by GHRH antagonists. The antitumor action of GHRH antagonists can be also indirect and exerted through the inhibition of GH secretion from the pituitary. This mechanism causes the suppression of the pituitary GH/hepatic IGF-1 axis [11, 12, 15, 33]. GHRH antagonists also have beneficial effects on cognition, reduction of amyloid plaque and Tau filaments and inflammation in the most advanced transgenic mouse model of Alzheimer disease [67]. In addition, we have reported reduction of dyslipidemia in rats with MIA-602 class of GHRH antagonists [59].

According to some studies of Kovari et al., apocrine epithelium, and apocrine carcinomas show GHRH-R positivity [68]. These results suggest that GHRH-R expression is associated with casting-type calcifications on the mammogram. Apocrine carcinomas seem uniformly positive for GHRH-R. Whether these findings could indicate a potential role of GHRH-antagonists in targeted treatment of breast cancer [68].

Previous studies have shown that GHRH antagonists, such as MZ-J-7-118, MZ-5- 156 and JMR-132, inhibited the growth of human experimental endometrial carcinomas both in vitro and in vivo [38]. The beneficial oncological effects of these antagonists in experimental cancer treatment can be attributed to the suppression of pituitary-hepatic IGF-I axis and the direct inhibition through the binding of GHRH antagonists to pituitary GHRH-R and/or their splice variants present on tumors [36].

Based on the evidence that GHRH antagonists were able to suppress experimental tumor growth and that a subset of endometrial carcinoma expressed receptors for GHRH, the application of powerful new GHRH antagonists could be useful for the treatment of this type of malignancy [36].

Recent studies also demonstrated a mechanism by which GHRH-R antagonists such as MIA-602 target SV1 and inhibit the tumor growth of esophageal squamous cell carcinoma mediated by SV1. These findings suggest that SV1 is a hypoxia-induced oncogenic promoter that can be a potential target of GHRH-R antagonists [3, 36, 38].

Malignant pleural mesothelioma (MPM) is an aggressive malignancy associated with exposure to asbestos, with poor prognosis and no effective therapies. GHRH, GHRH-R, and its main splice variant SV1 were found in all the MPM cell types examined. In vitro, MIA-602 and MIA-690 reduced survival and proliferation in MPM cell lines and primary cells and showed synergistic inhibitory activity with the chemotherapy drug pemetrexed [69]. In MPM cells, GHRH antagonists also regulated activity and expression of apoptotic molecules, inhibited cell migration, and reduced the expression of matrix metalloproteinases. These effects were accompanied by impairment of mitochondrial activity and increased production of reactive oxygen species. In vivo, s.c. administration of MIA-602 and MIA-690 at the dose of 5 µg/d for 4 weeks strongly inhibited the growth of MPM xenografts in mice, along with reduction of tumor insulin-like growth factor-I and vascular endothelial growth factor. Overall, these results suggest that treatment with GHRH antagonists, alone or in association with chemotherapy, may offer an approach for the treatment of MPM [69].

Very recent results, showing a marked incidence of GHRH and SV1 of GHRH-R in various neoplastic hematological and oncological disorders in children, support the merit of further investigation of GHRH-Rs as potential molecular targets for diagnosis and therapy [70]. This is in agreement with a previous report of Jimenez et al. [71] who demonstrated the presence of GHRH-R in three human acute myeloid leukemia (AML) cell lines KG-1a, K-562 and THP-1 and in nine specimens from patients with AML. Significant inhibition of cell proliferation in these cell lines following treatment with GHRH antagonist MIA-602 was found in vitro. In addition, treatment with MIA-602 of mice bearing tumor xenografts of these three human AML models, resulted in effective tumor growth inhibition [71].

Recently developed peptide antagonists of GHRH-R inhibit the growth of various cancer cell lines and have pre-clinical benefits against prostate cancer, inhibit human gastric cancer, induce apoptosis in retinoblastoma cells and suppress the growth of human malignant melanoma. However, no non-peptidic small molecules targeting the receptor have been reported. The molecular basis of GHRH binding to the extracellular domain still remains unknown, and therefore the potential to unravel it holds a great opportunity towards a new generation of drug candidates for cancer therapy [39].

## Data Availability

No datasets were generated or analysed during the current study.
